# Novel Approach to Meta-Analysis of Microarray Datasets Reveals Muscle Remodeling-related Drug Targets and Biomarkers in Duchenne Muscular Dystrophy

**DOI:** 10.1371/journal.pcbi.1002365

**Published:** 2012-02-02

**Authors:** Ekaterina Kotelnikova, Maria A. Shkrob, Mikhail A. Pyatnitskiy, Alessandra Ferlini, Nikolai Daraselia

**Affiliations:** 1 Ariadne Genomics Inc., Rockville, Maryland, United States of America; 2 Section of Medical Genetics, Department of Experimental and Diagnostic Medicine, University of Ferrara, Ferrara, Italy; Stanford University, United States of America

## Abstract

Elucidation of new biomarkers and potential drug targets from high-throughput profiling data is a challenging task due to a limited number of available biological samples and questionable reproducibility of differential changes in cross-dataset comparisons. In this paper we propose a novel computational approach for drug and biomarkers discovery using comprehensive analysis of multiple expression profiling datasets.

The new method relies on aggregation of individual profiling experiments combined with leave-one-dataset-out validation approach. Aggregated datasets were studied using Sub-Network Enrichment Analysis algorithm (SNEA) to find consistent statistically significant key regulators within the global literature-extracted expression regulation network. These regulators were linked to the consistent differentially expressed genes.

We have applied our approach to several publicly available human muscle gene expression profiling datasets related to Duchenne muscular dystrophy (DMD). In order to detect both enhanced and repressed processes we considered up- and down-regulated genes separately. Applying the proposed approach to the regulators search we discovered the disturbance in the activity of several muscle-related transcription factors (e.g. MYOG and MYOD1), regulators of inflammation, regeneration, and fibrosis. Almost all SNEA-derived regulators of down-regulated genes (e.g. AMPK, TORC2, PPARGC1A) correspond to a single common pathway important for fast-to-slow twitch fiber type transition. We hypothesize that this process can affect the severity of DMD symptoms, making corresponding regulators and downstream genes valuable candidates for being potential drug targets and exploratory biomarkers.

## Author Summary

Comparison of gene expression in diseased and normal tissue is a powerful tool of studying processes involved in pathogenesis and searching for potential drug targets and biomarkers of the disease's progression and treatment outcome. We have developed a novel approach for systematic knowledge-driven analysis of gene expression profiling data, which can suggest the underlying cause of the observed differential expression by identifying which expression regulators might be involved. These regulators can not only be the promising subjects of further investigation, but also potential drug targets, as normalization of their activity might alleviate some of the disease's symptoms. The targets downstream of suggested regulators can be proposed as exploratory biomarkers in disease treatment and prognosis. We used our approach to analyze public gene expression datasets of Duchenne muscular dystrophy – a progressive inherited disease in males. Some of the regulators and biomarkers that we found were already investigated in the context of DMD, while some of them were not yet studied and may be of interest for biological and clinical studies.

## Introduction

Microarray-based expression profiling is a widely used, quick and inexpensive method to obtain information about the specific diseases. A traditional approach when searching for drug targets or candidate biomarkers for a specific disease is to look for genes differentially expressed between the disease and appropriate “control” samples. Various techniques have been applied to find statistically significant differentially expressed genes, including classical statistical tests (e.g. t-test) and those specifically developed for microarray data analysis (Limma [1], SAM [2], shrinkage T-statistic [3] and other).

To get the deeper understanding of the disease mechanisms, the functional analysis of differential genes can be performed using a number of different methods [4]. Typically they rely on Gene Ontology (GO) – based annotation of genes. Common approach is to pre-select differentially expressed genes based on differential fold-change and/or p-value threshold, and find the statistically enriched GO groups using Fisher's exact test. More sensitive approaches are based on gene set enrichment analysis (GSEA [5], [6]) to avoid differential cut-off selection issue.

In addition to Gene Ontology, the protein-protein functional associations, regulatory or biochemical networks can also be used as a source of functional protein annotation in enrichment analysis [6], [7], [8]. More elaborated classification and functional annotation methods [9], [10] are usually applied to protein-protein networks only. The potential drawback of this kind of networks for the analysis of expression data is that they eventually skip the important transcriptional factors if they are not differentially expressed themselves. In this paper we used a proprietary literature-derived gene expression regulation network as a source of functional protein annotation. This global expression network consists of direct or indirect effects of a network node (protein) on expression of other genes [11]. Unlike conventional GSEA [5], [6], which uses predefined collection of gene sets, Sub-Network Enrichment Analysis (SNEA) algorithm, implemented in Pathway Studio® software [11], constructs comprehensive collection of gene sets from ResNet, a global literature-extracted protein-protein regulation network. The gene sets are constructed for each individual network node (“seed”) and consist of all its downstream expression targets only (star-like subnetworks).

The central idea of SNEA approach is that if the downstream expression targets of a “seed” are enriched with differentially expressed genes, then the “seed” is likely to be one of the key regulators of the differential expression changes, e.g. a transcription factor responsible for the observed changes in expression or an upstream member of signaling pathway [12]. This literature-driven approach connects differentially expressed genes to major implicated pathways and key expression regulators. In contrast to other methods that utilize the same idea of finding upstream network regulators using expression data [13], [14], SNEA allows identification of any potentially important protein (not obligatory a transcriptional factor) leading to the observed expression changes, even if its own expression doesn't change. It becomes possible because of the usage of ResNet database where all relations are taken from the literature only. Hence, there is no restriction on the protein type that can be considered as potential “seed”, provided that it is reported to influence each individual downstream gene expression.

We have applied this approach to study Duchenne muscular dystrophy (DMD) using publicly available gene expression profile datasets and identified a set of potential regulators and downstream biomarkers of DMD progression and severity.

Duchenne muscular dystrophy is an X-linked recessive muscular disorder, caused by mutations in the dystrophin gene (DMD) [15]–[17]. Affecting about 1∶3500 newborn males, it is the most common form of muscular dystrophies and the most common sex linked disease in males [18]. The underlying genetic cause of DMD is the presence of a variety of *DMD* gene mutations that result in dystrophin reduction/absence in skeletal muscle [17]. Lack of dystrophin has multiple unfavorable consequences to a muscle fiber (reviewed in [19]), leading to apoptosis or necrosis with subsequent inflammation and fibrosis at the site of damage. The process of muscle regeneration is also activated, but, in humans, with the course of the disease the repair capacity declines and becomes insufficient [20]. Muscle tissue is replaced with adipose and fibrous connective tissue [21].

The average life expectancy of DMD patients varies from late teens to early thirties, and can be improved by respiratory support [22], [23] and drug therapy [24]. Currently, there is no cure for DMD, but some treatments targeting the secondary consequences of dystrophin deficiency, such as muscle damage, necrosis, apoptosis and failure of regeneration, are already available for patients. Glucocorticoids, such as prednisone and deflazacort, are widely used to alleviate some of the disease's symptoms [25].

Several tests are used in diagnostics of DMD, including measurement of physical parameters, serum level of creatine kinase, genetic testing for DMD mutations and muscle biopsy to confirm the reduction in dystrophin content. More accurate, preferably non-invasive and biologically explainable markers are needed to predict prognosis, estimate disease's severity and progression. Also new biomarkers are required in treatment and clinical trials for DMD, where they can be used to monitor drug efficiency and choose optimal drug dose.

In order to identify potential drug targets along with corresponding biomarkers, we have searched for the consistent SNEA regulators and their downstream expression targets using publicly available differential gene expression profiles and literature-extracted expression regulation network from muscle biopsies of patients with DMD. Suggested workflow implies aggregation of the data from multiple datasets and elucidation of common mechanisms that underlie differential expression. Studying these mechanisms from the prospective of searching for new drug targets can provide valuable insights in both biological and medical research.

## Results/Discussion

### Workflow

**Figure 1:**
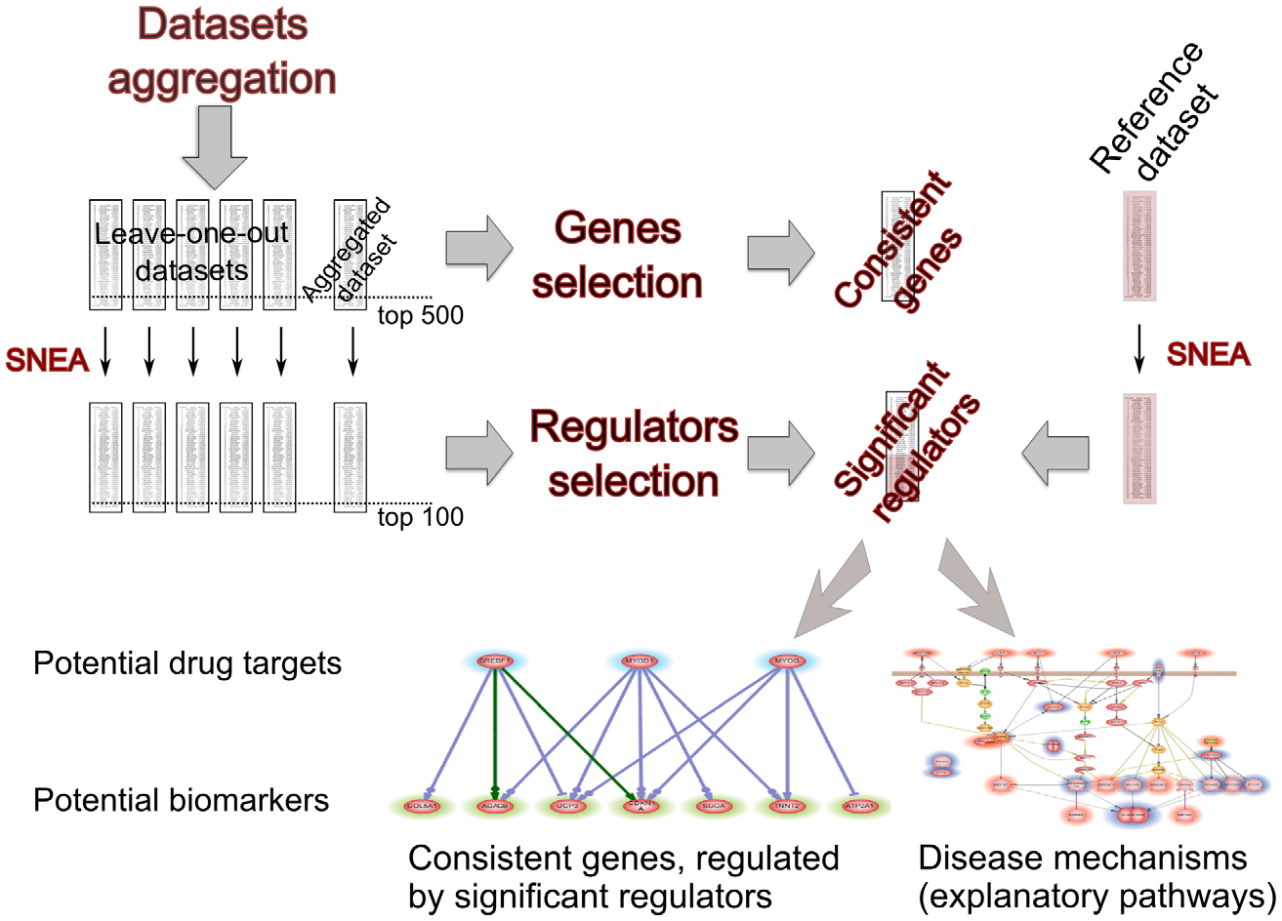
Overall workflow of the analysis. See corresponding section for detailed description.

The overall analysis workflow is presented in Figure 1. Five NCBI GEO DMD-related microarray expression profiles from muscle biopsies were aggregated according to the procedure described in Methods. To ensure robustness of our analysis we constructed five leave-one-out datasets each time aggregating four distinct experiments and omitting one out of total five available experiments. We also constructed single large dataset (referred to as “aggregated dataset”), where all five available microarray experiments were aggregated. Additional dataset (referred to as “reference dataset”) was constructed on the base of published meta-analysis [26], see Methods.

We performed SNEA with default parameters for each of the six datasets (five leave-one-out datasets plus aggregated dataset) and obtained six lists of 100 significant regulators. Regulators common for all six datasets were combined with regulators obtained by SNEA of reference dataset. This resulted in the list of 76 unique regulators, which can be viewed as potential drug targets. We also performed permutation test to ensure that this overlap is significant.

Next, we turned to selection of differentially expressed genes. For each of the 6 datasets (five leave-one-out datasets plus aggregated dataset) we performed gene ranking using combination of different methods (see Methods section). Then we identified genes which were present in top-500 lists for all six datasets. Out of all these consistently differentially changed genes, we have selected only those which were expression targets of selected consistent significant regulators. This produced a list of 140 candidate genes (105 over- and 35 under-expressed). These genes (potential biomarkers) have been sorted using the combination of expression rank in the aggregated dataset and the number of significant regulators as a score (see Methods section). We also manually evaluated top-20 up-regulated genes and top-10 down-regulated genes in respect to the supporting evidences from the available literature.

All analytical procedures were applied separately to over-expressed genes and under-expressed genes to look individually at processes and pathways activated and repressed in DMD.

### Significant regulators identified by SNEA

The significant regulators of up- and down- regulated differentially expressed genes from six datasets were cross-validated and only those identified in all datasets were selected for further analysis. They were combined with regulators obtained from the SNEA of the reference dataset to produce the final list of 76 unique significant regulators shown in Table 1 below. More information about these regulators can be found in Table S1.

**Table 1:** Consistent regulators of differentially expressed genes plus regulators from SNEA of reference dataset.

Function	Regulation	Regulators
Transcription factors	positive, negative	AML1-ETO, CCAAT factors, CIITA, CTCF, ESRRA, FOXI1, ING4, MEF2C, MYOD1, MYOG, NF-kB, NR1H2, NR1H4, NR4A2, PPARD, PPARGC1A, RUNX1, RUNX2, SCXA, SMAD, SMAD7, SREBF1, SREBF2, STAT1, TWIST1, ZEB1, ZFHX3
Cytokines and cytokines receptors	positive, negative	ADIPOQ, BMP2, CSF1, CSH1, CTGF, GCG, GH1, IFNG, IL13, IL4, IL6, IL6R, INS, LEP, PTH, TGF family, TGFB1, TGFB2, TNFRSF11B
Growth factors	positive	AGT, BMP2, CSF1, CTGF, FGF2, GF, IL4, IL6, TGF family, TGFB1, TGFB2
Hormones	positive, negative	ADIPOQ, AGT, CSH1, GCG, GH1, INS, LEP, PTH
MAPK	positive	MAPK, MAPK3
Extracellular matrix	positive	collagen type I, vitronectin
Inflammation and immune response	positive	allergen, CAMP, CCL2, CCR7, CIITA, CMA1, CXCL2, IFNG, IL13, IL4, IL6, IL6R, IRF1, NF-kB, STAT1, TGF family, TGFB1, TGFB2, TNFRSF11B, ZEB1
Regulation of metabolic processes	negative	ADIPOQ, ADRB3, AMPK, GCG, INS, LEP, NR1H2, PPARD, PPARGC1A, PRKAA2, SREBF1, SREBF2, TORC2, UCP2
TGFB-SMAD pathway	positive	BMP2, SMAD, SMAD7, TGF family, TGFB1, TGFB2
Muscle-specific factors	negative	MEF2C, muscle fiber, MYOD1, MYOG
Cell cycle	positive	CDKN1B, CTCF, ING4, SCXA
IFNG signaling	positive	IFNG, IRF1, STAT1
Renin-angiotensin system	positive	AGT, angiotensin II receptor
Chromatin modification	positive, negative	HDAC1, histone deacetylase inhibitor
Other	positive	alkaline phosphohydrolase, GJA1, LPL, MIRN29C, RHOA

#### Regulators of up-regulated genes

Overall, regulators of up-regulated genes correspond to the major processes that take place in dystrophic muscle, such as inflammation, fibrosis and muscle regeneration. Among regulators of up-regulated genes we can separate members of several known signaling cascades: NFKB, angiotensin signaling (AGT, functional class angiotensin II receptor, chymase (CMA1)), TGF signaling (functional class TGF family, TGFB1, TGFB2, BMP2, functional class SMAD, SMAD7), and interferon gamma signaling (IFNG, STAT1, IRF1), suggesting that these pathways may be disturbed in dystrophin-deficient muscle.

An indirect proof of our approach is the fact that some of our regulators were shown to contribute to the disease progression in DMD patients and animal models of DMD, such as mice (mdx) and golden retriever (GRMD). Mdx mouse is the most widely used model of DMD, although the pathology is much milder in these animals. GRMD is clinically more similar to an actual disease, due to the size of animals and severity of symptoms [27], [28]. According to PubMed at least 17 out of 37 SNEA-derived regulators of up-regulated genes are related to DMD in human or animal models. Moreover, several regulators were already tested as potential drug targets in mdx mice with generally positive outcome, suggesting that the rest of SNEA-proposed regulators also might be of interest. For example, there is strong evidence of NFKB pathway involvement in DMD progression [29], [30]. Blocking of NFKB was suggested as a potential therapy against DMD, as it stimulates regeneration and decreases necrosis in mdx mice [31], [32].

It was also shown, that members of angiotensin system are overexpressed in dystrophic muscles and that they may play role in subsequent activation of TGFB signaling cascade [33], observed in DMD patients [34], [35]. TGFB plays role in fibrosis and also in impaired muscular regeneration through inhibition of myogenic factors MYOG and MEF2D, and repression of myotubes formation [36]. Noteworthy, we found that another member of TGFB family, TGFBR2, was a consistently differentially expressed gene. Angiotensin II receptor and angiotensin converting enzyme were widely studied as drug targets in the context of DMD [37]–[39].

Role of TGFB1 was shown in humans, mdx mice and GRMD [40]. Recently TGFB1 was tested as a potential drug target and it was shown, that its inhibitors protect muscles of mdx mice from exercise induced damage and decrease fibrosis [41].

Activation of TGFB may by turn cause up-regulation of connective tissue growth factor (CTGF) [42] and vice versa [43], promoting fibrotic changes in dystrophin-deficient skeletal and cardiac muscles [40], [42], [43].

Functioning of histone deacetylases (HDACs) is affected by dystrophin deficiency, what can be reverted by HDAC inhibitors (reviewed in [44]

Activation of IFNG pathway may contribute to muscular regeneration, fibrosis, inflammation and antigen presentation [45]–[47]. The involvement of IFNG signaling in DMD was demonstrated in several publications: IFNG production was shown to be increased in lymph nodes [48] as well as transcriptional activity of its downstream target STAT1 in diaphragm muscles of mdx mice [49].

The level of another SNEA-derived regulator, FGF2, is also elevated both in mdx mice [50] and in serum of Duchenne patients [51]. FGF2 is involved in skeletal satellite cells activation and proliferation [52], and its blood level correlates with muscular regeneration in DMD patients and thereby it can be used as a biomarker of this process [53].

The role of transcription factor ZEB1 (zinc finger E-box-binding homeobox 1) in DMD hasn't yet been described in literature. ZEB1 inhibits muscular differentiation by blocking transcriptional activity of myogenic transcription factors, such as MEF2C [54]. Interestingly MEF2C is a SNEA-derived regulator of down-regulated genes. In addition ZEB1 synergize with SMAD and can regulate TGFB signaling [55]. As both myogenesis and TGFB signaling are affected in DMD, studying ZEB1 in the context of DMD may look promising.

Being one of the top up-regulated genes in aggregated dataset (rank 7, log-ratio 2.11) RUNX1 was also found as a significant regulator of up-regulated genes from reference dataset. To our knowledge there are no publications, establishing linkage between RUNX1 and DMD. RUNX1 may be relevant for the disease, as it is strongly induced in denervated muscles, where its proposed role is to protect disused myofibers from disorganization, autophagy and muscle wasting [56].

Taking into the account the strong literature support of described regulators significance we can suggest other SNEA-derived regulators as well as their functional protein partners for further investigations for the role of potential drug targets.

#### Regulators of down-regulated genes

Up-regulation of inflammation-related genes is the most prominent expression pattern in dystrophin-deficient muscle. Separation of down-regulated genes allows independent analysis of the processes potentially repressed under this condition.

**Figure 2:**
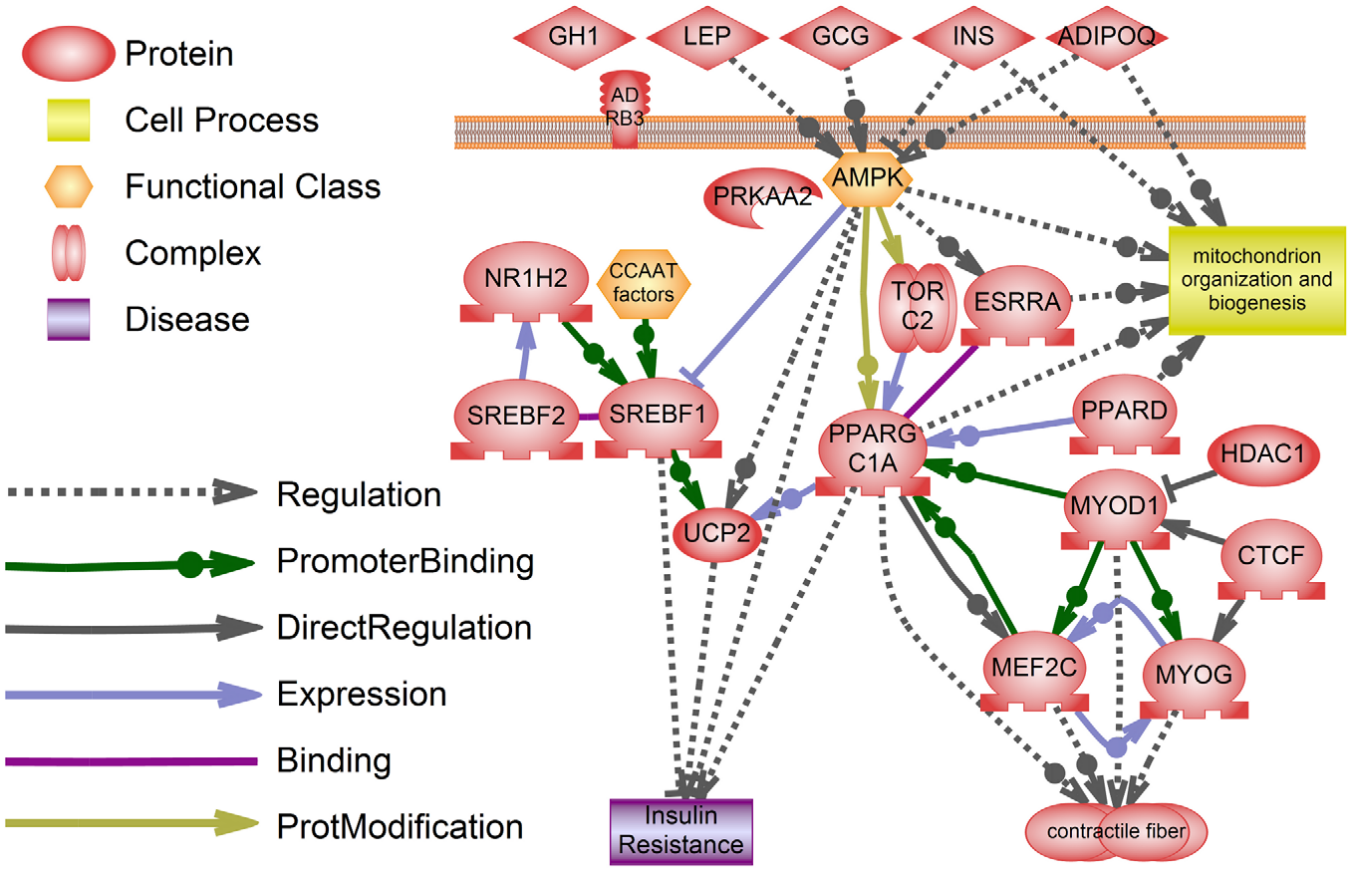
Regulators of down-regulated genes. Most of SNEA-derived regulators of down-regulated genes regulate the processes related to myotube formation, fast-to-slow fiber type switch (including changes in myofiber composition, mitochondria content and insulin sensitivity) and metabolic changes in DMD affected muscles. Relations are described in text. Catalytic subunit of AMPK, PRKAA2, is shown next to AMPK. Functional class - class of proteins, such as enzyme families. Complex - a group of two or more proteins linked by non-covalent protein-protein interactions. Expression - protein members of one class regulate expression of proteins in another class. DirectRegulation - protein members of one class bind and regulate proteins in another class. Regulation - protein members of one class indirectly regulate proteins in another class. ProteinModification - protein members of the regulator class phosphorylate or otherwise modify proteins in the target class. PromoterBinding - protein members of one class bind promoters of genes encoding proteins in another class.

Among proteins that regulate expression of negatively regulated genes there is a group of factors working synergistically in a number of processes crucial to a muscular physiology, e.g. muscle remodeling and myogenesis (see Figure 2, representing some of the relations between regulators of down-regulated genes).

In response to the changing environmental and physiological demands myofibers can significantly alter the gene expression to adapt to the current needs. It happens through the switch between slow and fast fiber types that differ in their size, metabolism and contractile function, in a process of muscle remodeling. Slow-twitch fibers are rich in mitochondria content, have oxidative metabolism and are resistant to fatigue. Fast-twitch fibers are glycolytic and function in quick contractions (reviewed in [57]). DMD preferentially affects fast-twitch myofibers, while slow-twitch fibers show less damage [58]. One of the proposed reasons of higher slow fibers' survivability is up-regulation of utrophin, a dystrophin homolog that can function as a partial replacement for dystrophin [59].

Several factors that were obtained by SNEA of down-regulated genes play role in muscle remodeling (e.g. PPARGC1A, PPARD, AMPK, TORC2, MEF2C, MYOG, MYOD). They coordinate mitochondria biogenesis, metabolic and transcriptional changes that are necessary for transition to a slow-twitch muscle type. Some of them, such as PPARGC1A and its activator PPARD [60], were already studied in the context of DMD. It was known that activation of PPARGC1A and PPARD by over-expression or treatment with agonists ameliorates disease's symptoms in mdx mice by promoting slow fibers formation, up-regulation of utrophin and enhancing neuromuscular junction program [61], [62]. The role of PPARGC1A was also demonstrated in GRMD, where it was shown that PPARGC1A along with its targets is dramatically reduced [63]. Recently the role of another regulator predicted by our SNEA analysis AMPK, was also confirmed in mdx mice. It was shown that activation of AMPK by its agonist, AICAR, enhanced oxidative capacity, elicited fast-to-slow fiber type transition, up-regulated utrophin expression and increased sarcolemmal integrity [64].

AMPK, PPARGC1A, PPARD as well as other factors important for fast-to-slow twitch fiber transition activate in response to exercise, therefore a group of compounds, simulating the effect of physical exercise, called exercise mimetics, can be suggested as potential drugs to be tested in mdx mice. Some exercise mimetics were already successfully tried in mdx mice (e.g. GW1516, AICAR, resveratrol [62], [64], [65]). Some of the other compounds known to stimulate the respective regulators can also be suggested to improve symptoms in dystrophin deficiencies, e.g. metformin, acadesine, phenormine, berberine (AMPK stimulators), bezafibrate and GW0742 (PPARD stimulator), pioglitazone and forskolin (PPARGC1A stimulator), SRT1720 (a more effective stimulator of SIRT1, than resveratrol).

Interestingly, prednisone, a glucocorticoid that is used in the therapy of DMD has an opposite effect on muscle fiber type, decreasing the number of slow-twitch fibers [50].

Another group of significant regulators, such as TORC2 and UCP2, have not yet been linked to Duchenne muscular dystrophy, but they are known to regulate mitochondrial biogenesis, which takes place during muscle remodeling (reviewed in [66], [67]). We can hypothesize, that mitochondria biogenesis is repressed in dystrophic muscle, as 34 out of 191 consistently down regulated differentially expressed genes are expressed in mitochondria (e.g. 6 NADH dehydrogenase subunits, 4 mitochondrial ribosomal proteins, components of respiratory chain and tricarboxylic acids cycle).

All above-mentioned factors work synergistically during formation of a slow-twitch myofiber. AMPK activates and up-regulates PPARGC1A [68], [69] and attenuates the gluconeogenic program by blocking TORC2 nuclear accumulation [70], [71]. TORC2 is also able to promote mitochondrial biogenesis and enhance oxidative capacity in muscle cells by stimulating PPARGC1A transcription and up-regulation of ESRRA [67], transcription factor known to be involved in mitochondrial biogenesis and myotube formation [72]. UCP2 is a downstream target of PPARGC1A [73]. Myogenic factors MYOG, MYOD and MEF2C were shown to bind PPARGC1A promoter at the late stages of muscle differentiation [74], [75].

The process of muscle remodeling is connected to the change in insulin sensitivity. It was shown, that fast-twitch myofibers are more insulin resistant, while slow-twitch myofibers are more insulin sensitive [76]. Interestingly insulin is one of the significant regulators of down-regulated genes derived from analysis of reference dataset, as well as glucagon and adipokines, leptin and adiponectin. The presence of adipokines among regulators of gene expression in DMD can be explained by metabolic and histological changes in dystrophic muscle.

Three myogenic factors: MYOD, MYOG and MEF2C, co-acting during muscle development ([77], reviewed in [78]) were shown to be significant regulators of down-regulated genes in aggregated dataset. Many of the aspects of their involvement in DMD have been already studied, and our results just confirm their importance in DMD pathogenesis. For example, lack of a master regulator of skeletal muscle gene expression program MyoD was shown to result in a significant increase in myopathy's severity and premature death in mdx mice due to the decreased regeneration ability [79]. MyoD impaired activity in dystrophin-deficient muscle can be caused by activation of NFkB and IFNG pathways that result in MyoD destabilization [80]. Deletion of another myogenic factor, MYOG, on the contrary benefits mdx mice by improving fatigue resistance [81]. Both MYOG and MEF2C are regulated by MYOD. Interestingly, one of the regulators of down-regulated genes is transcription factor CTCF, found recently to be a modulator of MyoD and MyoG activity during myogenesis [82]. HDAC1 is also involved in regulation of myogenic program by blocking MYOD-mediated transcription [83].

As the set of described regulators reflects the impairment of the same group of processes and 6 of 15 regulators were already mentioned in the context of DMD and even tested as drug targets, we can suggest, that the others, such as TORC2, can also be considered from this point of view.

### Selection of differentially expressed genes consistent between 5 datasets

**Figure 3:**
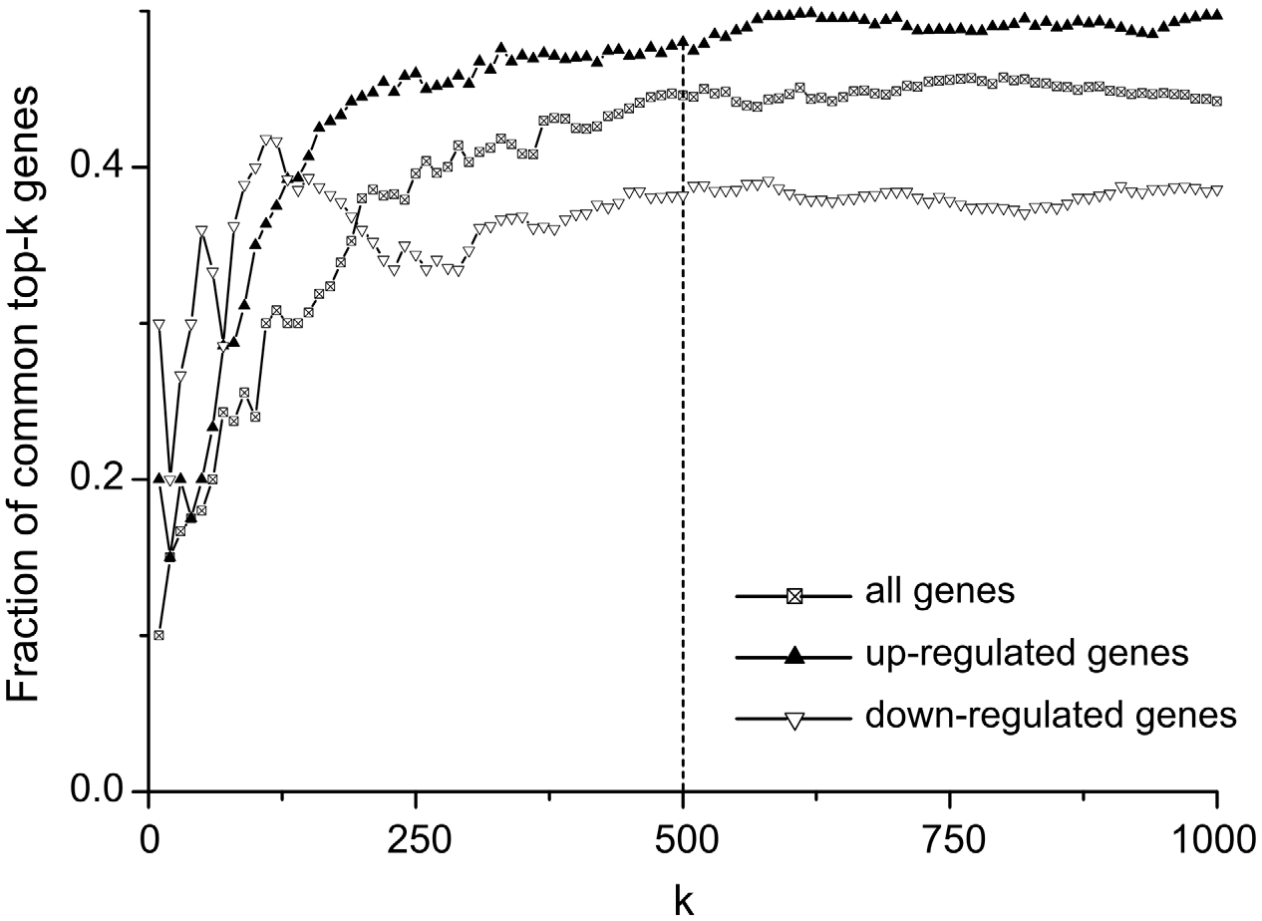
Fraction of common genes in top-k rankings for different types of gene expression. For each of six datasets and for each type of regulation gene ranking procedure was performed and overlap between six top-k lists was calculated. Fraction of common genes in top-k reaches saturation for k roughly equal to 500, hence adding more genes will not increase overlap between six rankings.

We have selected genes, which were consistently differentially expressed in six datasets (one aggregated dataset and five leave-one-out datasets). The fold-change threshold was established by analyzing fraction of genes present in all six top-*k* rankings for varying *k*, Figure 3. As can be seen, fraction of common genes in top-*k* rankings for different types of gene expression reaches a plateau for *k* roughly equal to 500. This means, that adding more genes will not increase percentage of overlap between different gene rankings. Hence we limited our analysis to top-500 differentially expressed genes for different types of regulation. The percentage of consistent genes in top-*k* of all datasets is about 40% (Figure 3). It means that analysis of differentially expressed genes from a single dataset can potentially lead to 60% of false positives. To increase reproducibility of obtained results we focused on the genes, presented in all six top-500 rankings.

From the top 500 up-regulated genes in aggregated dataset we have selected 240 genes also present among top 500 up-regulated in all 5 leave-one-out datasets. Similarly, from the top 500 down-regulated genes in aggregated dataset we have selected 191 genes also present among top 500 down-regulated in all 5 leave-one-out datasets. These two lists were combined into a single list of 431 consistently up/down regulated differential genes. We performed Fisher exact test to find significantly enriched categories from Gene Ontology, corresponding to biological processes. Results, presented in Table 2, in general reflect known changes that take place in affected muscles: up-regulated genes are commonly associated with inflammation and immune response, apoptosis and wound healing; down-regulated genes – with metabolic processes and muscle contraction.

**Table 2:** Gene Ontology groups enriched by consistent differentially expressed genes.

GO Process	Number of genes	p-value
**Up-regulated genes**		
cell adhesion	23	1.92E-09
immune response	20	6.8E-08
proteolysis	13	0.00193
apoptosis	12	0.001376
negative regulation of cell proliferation	10	0.00025
inflammatory response	10	0.000111
cell motion	10	3.76E-08
heart development	9	1.08E-05
skeletal system development	9	2.48E-06
wound healing	9	3.45E-09
**Down-regulated genes**		
carbohydrate metabolic process	16	2.94E-11
metabolic process	14	0.000619
oxidation reduction	12	0.00102
modification-dependent protein catabolic process	11	0.000211
glycogen metabolic process	9	2.16E-12
muscle contraction	8	1.01E-07
response to hypoxia	7	0.000119
electron transport chain	7	6.87E-06
nervous system development	6	0.040331
response to drug	6	0.00847

Biological processes from Gene Ontology associated with consistently differentially expressed genes were found by applying “Find groups enriched with selected entities” tool embedded in Ariadne Pathway Studio to the list of 431 genes. Resulting significant (p-value<0.05) biological processes were sorted by number of genes involved in a process. Top 10 processes are shown.

Genes were further analyzed in order to evaluate their quality as biomarkers. A promising biomarker should be easily detected and correspond to a DMD-related process (e.g. muscle biology, fibrosis, inflammation) or DMD-related condition (e.g. dilated cardiomyopathy). We used a proprietary Ariadne DiseasesFX Database, which contains literature-extracted information about various types of relations between genes and diseases as well as data on presence of gene products in biofluids and among secreted proteins. We also made use of Ariadne ResNet 7 and Muscle Biology Gene Ontology, see Methods. Associations between 431 consistently up/down regulated genes and DMD-related processes and conditions are depicted in Table S2.

### Consistent differentially expressed genes downstream from significant regulators

Out of 431 consistently changed genes, we have selected only those which are expression targets of significant regulators, selected using the above procedure. This produced a list of 140 candidate genes (35 down-regulated, 105 up-regulated) that have been finally sorted using combination of rank in aggregated dataset and number of significant regulators (see Methods). Most of them correspond to the processes of development and regeneration, immune response, response to glucocorticoids, hypoxia and extracellular matrix organization.

Top-ranked 20 positive and 10 negative genes have been individually analyzed using biological information available from scientific literature (PubMed). Mainly they are connected to fibrosis, inflammation, energy metabolism and other processes known to be affected in DMD. It was found that 12 out of these 30 were previously reported as related to muscle processes/disorders, the fact that can be considered as a proof of concept, providing the possibility to suggest new possible biomarker candidates on the base of suggested procedure.

In summary, this study demonstrates the possibility to decipher regulatory mechanisms of the specific disease (Duchenne dystrophy here) along with corresponding exploratory biomarkers on the base of multiple microarray data meta-analysis only. A lot of predicted expressional regulators are known to be involved in DMD, suggesting that others will also be verified hereafter. This means that all of the proposed regulators can be considered for further drug discovery, whereas their consistently differentially expressed downstream genes can serve as exploratory biomarkers with implicated mechanistic models.

## Methods

### Source data

All available microarray datasets of human DMD with more than 10 samples (total 5 datasets, see Table 3) were downloaded from NCBI GEO database [http://www.ncbi.nlm.nih.gov/geo/]. For each probeset intensity values were log-transformed and normalized to zero mean and unit variance. Missing data were imputed using K-nearest neighbor method with k = 10.

**Table 3:** GEO datasets used for the meta-analysis.

GEO ID	Platform	Description	Source	Reference
GDS 214	custom Affymetrix	4 healthy, 26 DMD	Muscle	[87]
GDS 563	Affymmetrix U95A	11 healthy, 12 DMD	Quadriceps Muscle	[88]
GDS 1956	Affymetrix U133A	18 healthy, 10 DMD	Muscle	[84]
GDS 2855	Affymetrix U133B	20 healthy, 10 DMD	Muscle	[84]
GDS 3027	Affymetrix U133A	14 healthy, 23 DMD	Quadriceps Muscle	[89]

### Reference dataset

We have also utilized data presented in [26], where the lists of up- and down-regulated genes were extracted from research papers, related to skeletal muscle development and pathologies. We limited this dataset to studies of DMD or mdx mice resulting in total 2227 genes which were reported to be differentially expressed in at least in one paper prior to December 2005. For these genes we generated a pseudo-expression dataset for further analysis similar to the standard microarray experiment. If gene was reported to be up-regulated, the gene was assigned a positive value equal to corresponding number of supporting studies; if gene was reported to be down-regulated, the assigned value was negative.

### Dataset aggregation (gene ranking)

To combine the data from different datasets, we performed the following aggregation procedure. For each probeset we calculated within-dataset log-ratio, two-sample Welch's t-test, Wilcoxon rank sum test and area under ROC curve. If gene on a chip was represented by two or more probesets, we selected the probeset with the least p-value for Wilcoxon rank sum test. We also calculated several other statistics, using popular methods designed specifically for microarray data: limma, SAM and shrinkage T-statistic. Limma, Linear Models for Microarrays [1], [84], is based on a Bayesian hierarchical model for posterior odds of differential expression. SAM, Significance Analysis of Microarrays, was proposed in [2]. Shrinkage T-statistic stabilizes the variances in the denominator via a James-Stein approach [3].

Finally, we have combined the results from different experiments to generate the single “differential” rank for each gene. Separate gene rankings were obtained for nine measures: log-ratio, Welch's t-statistic and corresponding p-value, Wilcoxon's W-statistic and corresponding p-value, AUC, limma, SAM and shrinkage T-statistic. We used Fisher's method to combine p-values of the same type [85]; values of other statistics were averaged for each gene. The final gene rank *R* was calculated as mean of the ranks from all methods. Each gene was also assigned a single differential log ratio value calculated as an average differential log-ratio from 5 original gene expression datasets.

In order to ensure reproducibility of obtained results, we performed a procedure, analogous to leave-one-out cross-validation: we constructed additional datasets each time aggregating 4 distinct microarray experiments out of total 5 available experiments. Thus we obtained 5 leave-one-out datasets where each microarray experiment was omitted. We also built one large dataset, where all 5 available microarray experiments were aggregated. All subsequent analyses were performed for resultant 6 datasets and the results were cross-validated as further described.

### Sub-Network Enrichment Analysis

For functional analysis of high-throughput data on the level of potential regulators we used Sub-Network Enrichment Analysis (SNEA) algorithm, implemented in Pathway Studio software [11].

SNEA is a variation of gene set enrichment analysis algorithm, but unlike GSEA [5], [6] that uses predefined gene sets, SNEA utilized sub-networks to construct gene sets on the go. Here, each subnetwork consists of a node (mainly protein or class of proteins – “functional class”) in ResNet and all its expression downstream targets which are automatically derived from the literature. Global expression network includes direct (i.e. transcriptional factor A_1_ is reported in the literature to regulate specific gene B_1_) and indirect (i.e. growth factor A_2_, that can activate specific signaling pathway results to the change of downstream gene B_2_ expression) relations A_i_->B_i_. For each subnetwork seed SNEA considers all its expression targets as a gene set that is used for the classical GSEA (Mann-Whitney or Kolmogorov-Smirnov statistical tests).

Thus, SNEA determines the activity of expression regulators based on the differential expression of its targets and favors (assigns lower p-value) those of them which have more significant expression changes downstream.

We performed the SNEA in Pathway Studio with the default parameters: Sub-Network type: gene expression, Mann-Whitney test, p-value<0.05, number of regulators <100 for all log-ratio values (DMD vs. control) from the 6 aggregated datasets. The consistency of default parameters has been tested using 10 permutation tests. It has been shown, that the rate of significant SNEA seeds accidentally found in SNEA results applied to randomized experiment is less than 5%, which is in agreement with default p-value cutoff 0.05. For the reference dataset we ran SNEA with the same parameters using number of studies which reported gene to be differentially expressed. All enrichment algorithms were applied separately to over-expressed and under-expressed genes.

### Final gene sorting

The final sorting of the differentially expressed genes have been done using the following score
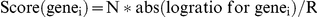
where *N* – number of significant regulators upstream of the *i*-th gene and *R* –gene rank in aggregated dataset resulted from expression data analysis only.

### Software and databases

Most computations were done using R [http://www.r-project.org/] and BioConductor [http://www.bioconductor.org/]. Values of limma, SAM and shrinkage T-statistic were computed using GeneSelector package [86].

Sub-Network Enrichment Analysis was performed using Pathway Studio 7.1 from Ariadne Genomics along with ResNet 7, database storing literature-derived network of biological relations [http://www.ariadnegenomics.com/]. Proprietary Ariadne DiseasesFX database was used for evaluation of gene quality as disease biomarker [Table S2], and ChemEffect [12] was used for studying drugs, related to the regulators of interest.

Muscle Biology Gene Ontology [http://wiki.geneontology.org/index.php/Genes_Involved_in_Muscle_Biology] was used to select genes associated with muscle-related processes.

## Supporting Information

Table S1 **Consistent regulators of differentially expressed genes.** Table contains description of SNEA-derived regulators (name, description, Entrez Gene ID); information whether regulator affects expression of up- or down-regulated genes, number and names of datasets, where regulator was found as a significant one; number of downstream consistently differentially expressed genes (see Table S2); rank in aggregated and reference datasets; information whether regulator was already mentioned in PubMed publications related to DMD.
(XLSX)

Table S2 **Consistently differentially expressed genes.** Table contains a list and description of consistently differentially expressed genes from aggregated dataset (description, Entrez Gene ID), their rank and log ratio, number of consistent regulators (see Table S1), regulating gene expression, association with DMD-related processes and conditions (from Ariadne DiseaseFX and ResNet7, Gene Ontology, Muscle Biology Gene Ontology).
(XLSX)
